# Transcription of the human and rodent *SPAM1 / PH-20 *genes initiates within an ancient endogenous retrovirus

**DOI:** 10.1186/1471-2164-6-47

**Published:** 2005-04-01

**Authors:** Catherine A Dunn, Dixie L Mager

**Affiliations:** 1 Terry Fox Laboratory, BC Cancer Agency, Vancouver, Canada; 2 Department of Medical Genetics, University of British Columbia, Vancouver, Canada

## Abstract

**Background:**

Sperm adhesion molecule 1 (SPAM1) is the major mammalian testicular hyaluronidase and is expressed at high levels in sperm cells. SPAM1 protein is important for penetration of the cumulus cell layer surrounding the ovum, and is also involved in zona pellucida binding and sperm intracellular signalling. A previous study had identified *SPAM1 *as one of the many human genes that initiate within a transposable element.

**Results:**

Examination of the human, mouse and rat *SPAM1 *loci revealed that transcripts initiate within the *pol *gene of an endogenous retrovirus (ERV) element. This is highly unusual, as all previously identified ERV-initiated cellular gene transcripts initiate within the viral long terminal repeat promoter. The *SPAM1 *locus therefore represents an example of the evolution of a promoter from protein-coding sequence. We have identified novel alternative promoter and splicing variants of human and murine *SPAM1*. We show that all transcript variants are expressed primarily in the testis and are predicted to encode identical proteins.

**Conclusion:**

The testis-specific promoters of the human and mouse *SPAM1 *genes are derived from sequence that was originally part of an ERV *pol *gene. This represents the first known example of an ERV-derived promoter acting in a gender-specific manner.

## Background

Sperm adhesion molecule 1 (SPAM1, also known as PH-20) is a member of a family of at least six mammalian hyaluronidases. The genes encoding these enzymes cluster in two groups of three – *SPAM1*, *HYAL4 *and *HYALP1 *(a pseudogene) on human chromosome 7q31, and *HYAL1*, *HYAL2 *and *HYAL3 *on human chromosome 3p21 [1,2]. The orthologous mouse genes form similar clusters at syntenic chromosomal locations [1]. This suggests that two single-gene duplications, followed by a small segmental duplication, occurred before the divergence of human and mouse approximately 80 million years ago.

HYAL4 exclusively degrades chondroitin. In contrast, HYAL1, HYAL2, HYAL3 and SPAM1 hydrolyze hyaluronic acid, with different substrate size preferences and tissue specificities [1-3]. Expression of *SPAM1 *has been unanimously reported in the testis in various species (reviewed in [1,4]). Expression has also been detected in the human epididymis, vas deferens, prostate and placenta [2,5] and the murine epididymis, kidney, uterus, vagina and oviduct [6-8]. Expression of *SPAM1 *has not been detected in the human female reproductive tract [2,9].

SPAM1 has various functions in fertilization. A catalytic domain has been shown to degrade hyaluronic acid [10,11]. This molecule is a major extracellular matrix component of the cumulus cell layer that surrounds the ovum, and SPAM1 has been shown to remove this cumulus layer *in vitro *[12]. SPAM1 has hyaluronic acid and zona pellucida binding regions that are distinct from its catalytic domain [13,14] and is also involved in an intracellular signalling pathway in sperm cells upon binding to the zona pellucida [4,15,16].

The role of murine SPAM1 in fertilization has been investigated using a knockout mouse line. Sperm from *Spam1 *^-/- ^mice showed a delay in the removal of the cumulus cell layer and fertilization *in vitro*. Surprisingly, however, *Spam1 *^-/- ^males showed normal *in vivo *fertility rates and sired normal-sized litters [17]. Sperm from *Spam1 *^-/- ^mice maintained 40% of the wild-type level of hyaluronidase activity, while protein expression assays indicated the presence of a second hyaluronidase in these cells [17]. This was unexpected, as SPAM1 was thought to be the only testicular hyaluronidase. These results may be explained by recent evidence that the murine orthologue of the human *HYALP1 *pseudogene has an intact ORF and is expressed in mouse testis [1,3,18], and that a seventh hyaluronidase, *Hyal5*, may exist in mouse, but not in human [3,18]. There may therefore be some redundancy among murine testicular hyaluronidases that explains the fertility of *Spam1 *^-/- ^mice. In this case, it remains likely that SPAM1 is an essential protein in human fertilization.

Little is known about the transcriptional regulation of *SPAM1*. A non-consensus cAMP response element (CRE) in the murine *Spam1 *promoter bound the testis-specific CRE modulator (CREM) protein and was involved in activation of *Spam1 *transcription *in vitro *[19]. In addition, *Spam1 *expression was abolished in CREM-deficient mice [19]. Various other putative transcription factor binding sites have been identified in the human, mouse and rat *SPAM1 *promoters [5-7,19,20]; however, the sites are generally non-consensus and have not yet been shown to be functional. The restricted developmental and spatial expression of SPAM1 [5,7,21], as well as the unique transcriptional mechanisms employed during spermatogenesis (reviewed in [22]), may render *SPAM1 *unamenable to traditional methods of transcription and promoter analysis.

In a previous study by our group, *SPAM1 *was identified as one of the many human transcripts that contain transposable element (TE) sequence [23]. TEs include long and short interspersed nuclear elements (LINEs and SINEs), DNA transposons, and endogenous retroviruses (ERVs). TEs are extremely common in the human and mouse genomes, and together contribute 45% and 40% of the total sequence, respectively [24,25]. Many human and mouse gene transcripts contain TE sequences in their untranslated regions (UTRs) [23,26,27]. TEs also contribute to the transcriptional regulation of many genes. The antisense LINE1 promoter and the long terminal repeat (LTR) promoters of ERVs are known to participate in the tissue-specific expression of various host genes [28-30]. Through bioinformatic analysis, human and mouse *SPAM1 *transcripts were predicted to initiate within an antisense ERV [23], indicating that this gene may represent another example of transcriptional regulation by a TE.

In this study, we show that the first exons and proximal promoter regions of the human and rodent *SPAM1 *genes are derived from an ERV1 *pol *coding region, and identify novel alternative promoters and splicing variants of the gene. We show that the human and mouse ERV-derived promoters are largely testis-specific, and discuss the implications of ERV insertion on the evolution of transcriptional regulation at this locus.

## Results

### The human *SPAM1 *gene initiates within an ERV1 *pol *region

A recent study by our group used bioinformatic methods to investigate the contribution of TEs to human and mouse gene transcripts [23]. That study determined that 3.1% of human RefSeq genes initiate within a TE sequence, indicating that these genes are candidates for transcriptional regulation by TEs. One example identified in this way was the *SPAM1 *gene, where the 5'-terminus was found to map within an antisense ERV element. We have now investigated this locus in more detail.

**Figure 1:**
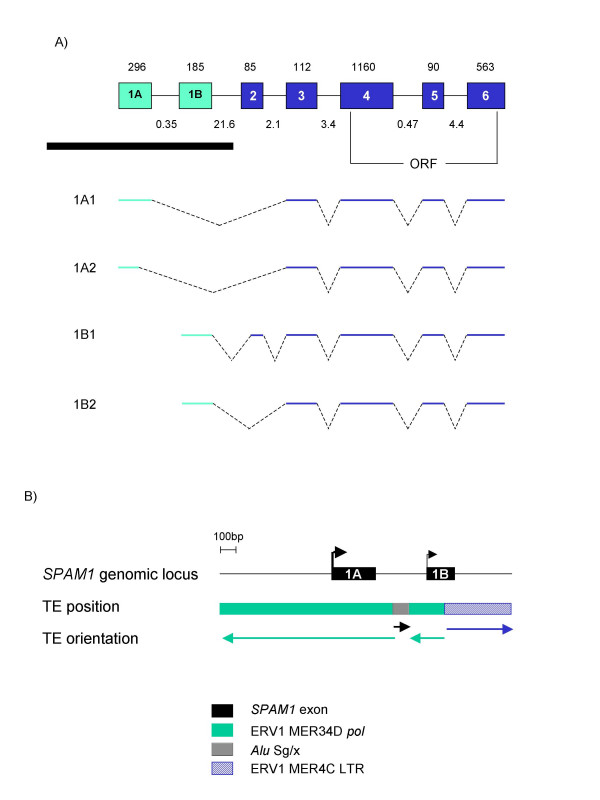
**Genomic structure of human *SPAM1*. **(A) Overview of the *SPAM1 *locus. Exons are boxed and numbered, with the size in bp shown above. Intron sizes in kb are shown below. The approximate positions of the *SPAM1 *ORF start and stop codons are indicated. The four *SPAM1 *splicing variants are shown schematically below. ERV-derived sequences are shown in green, with other sequences in blue. The diagram is not to scale. A black bar indicates the region shown in more detail in Figure 1B and 2. (B) The positions of *SPAM1 *alternative first exons 1A and 1B with respect to TE sequences. Exons are represented by solid black boxes. Bent arrows indicate the position and relative usage of the 5'-most transcription start site for each exon. TEs are represented by the colored boxes below; arrows indicate the orientation of each TE.

**Figure 2:**
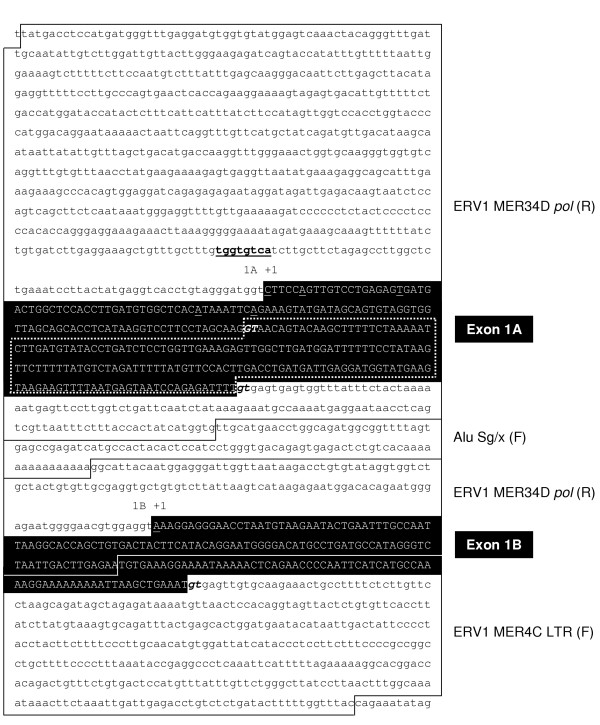
**The sequence and position of *SPAM1 *exons 1A and 1B with respect to TEs. **The sequence shown corresponds to human chromosome 7 co-ordinates 123158485 to 123160464 in the UCSC genome browser (May 2004 release). Solid lines define the boundaries between different TE sequences. The class and orientation (F, forward; R, reverse) of each TE are given on the right hand side. Exon sequences are shown in upper case reverse type. The dotted white line frames the portion of exon 1A included in splicing variant 1A1, but not variant 1A2. The transcription start sites identified by 5'-RACE are underlined, and splice donor sites are shown in bold italic type. A non-consensus CRE is shown in bold underlined type.

We used the University of California at Santa Cruz (UCSC) genome browser at  to more closely examine the genomic region surrounding the first exon of *SPAM1*. The region bears the hallmarks of multiple TE insertions into older, pre-existing repetitive elements, resulting in a "patchwork" effect of fragmented TEs from different families (Figure 1B, 2). The previously described *SPAM1 *transcriptional start site (nucleotide +40 in exon 1A) mapped within an antisense ERV1 element of the MER34 family (Figure 1B, 2). Surprisingly, the ERV sequence was derived not from an LTR, but rather from an internal retroviral region. This is counter to all previously described examples of transcription initiation within an ERV. To confirm this RepeatMasker annotation, we performed a BLAST homology search of the translated retroviral sequence against a protein database. This analysis confirmed that *SPAM1 *transcripts initiate within a fragment of the ERV1 *pol *gene.

### The alternative promoters and splicing variants of *SPAM1*

We performed 5'-rapid amplification of cDNA ends (RACE) to confirm the position of the *SPAM1 *transcriptional start site. Since expression of *SPAM1 *is confined largely to the testis, we used human testis RNA for this analysis. Sequencing of 5'-RACE clones identified two alternative first exons of *SPAM1 *(Figure 1, 2). We have designated the upstream, previously-identified first exon as exon 1A, and the novel downstream first exon as exon 1B. Exon 1A is wholly derived from the antisense ERV1 *pol *region. Exon 1B initiates within a different fragment of the same *pol *gene, but terminates within a sense orientation LTR of the ERV1 MER4C family (Figure 1B, 2). Transcripts containing both alternative first exons spliced into the same downstream exons; the *SPAM1 *ORF begins in exon 4, and is therefore not affected by alternative promoter usage (Figure 1A).

Multiple transcription start sites were identified within exon 1A, at position +1, +6, +20 and +51 (Figure 2). We also identified a splicing variant of exon 1A, with variant 1A2 using a splice donor site at position +118. Use of this alternative splice site resulted in a truncated 117 bp first exon, as opposed to a full-length size of 296 bp for variant 1A1 (Figure 1A, 2). In contrast, a single transcription start site and no splicing variants were observed for exon 1B. However, some transcripts initiating within exon 1B contained a novel alternatively-spliced 85 bp exon (Figure 1A). The sequences of all human and murine *SPAM1 *splicing variants have been deposited in GenBank with accession numbers AY920278 – AY920283.

### Both ERV-derived promoters are male-specific

**Figure 3:**
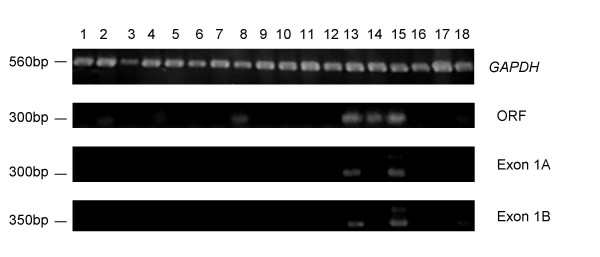
**Detection of *SPAM1 *transcripts by RT-PCR. **Primer pairs specific for *GAPDH*, the *SPAM1 *ORF, and *SPAM1 *transcripts initiating within exon 1A or 1B were used in RT-PCR assays. Assays were carried out on cDNAs derived from a range of normal human tissues: 1, brain; 2, heart; 3, kidney; 4, liver; 5, lung; 6, bone marrow; 7, colon; 8, small intestine; 9, spleen; 10, stomach; 11, thymus; 12, mammary gland; 13, prostate; 14, muscle; 15, testis; 16, uterus; 17, spinal cord; 18, placenta. Approximate molecular weights are indicated on the left.

To verify the expression patterns of the *SPAM1 *alternative promoter and splicing variants, we performed non-quantitative RT-PCR on a panel of RNAs derived from normal human tissues. As shown in Figure 3, expression of transcripts containing *SPAM1 *ORF sequence was detected in the testis, as well as the heart, small intestine, prostate, muscle and placenta. Primers designed to amplify both exon 1A splicing variants detected transcripts only in the testis and prostate, while exon 1B-specific transcripts were detected in the testis, prostate, and to a lesser degree in the placenta. The smaller of the two splicing variants was predominant for both promoters; this may be due to an amplification bias introduced by the PCR.

**Figure 4:**
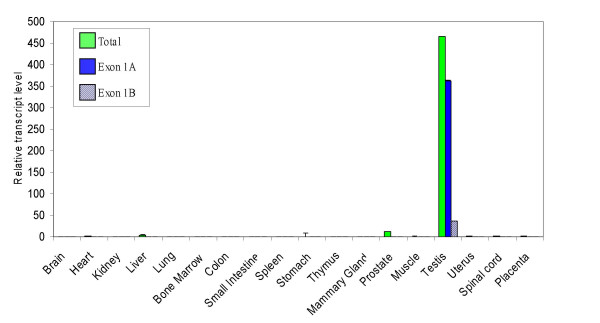
**Quantitative analysis of *SPAM1 *expression in normal human tissues. **Primers were used in real-time RT-PCR assays to amplify transcripts specific for *GAPDH *and total *SPAM1 *transcripts, and, in testis only, for *SPAM1 *transcripts initiating in exon 1A and 1B. Green bars represent the relative abundance of total *SPAM1 *transcripts normalized to *GAPDH*. Solid and hatched blue bars represent the contribution of exon 1A and 1B transcripts, respectively, to total *SPAM1 *expression. All bars represent the mean of four independent assays ± standard deviation.

We next used real-time RT-PCR to quantify the level of *SPAM1 *transcripts and the contribution of each alternative promoter to total gene expression. Primers annealing to exon 4 and exon 5, common to all *SPAM1 *transcripts (Figure 1A), were used to determine the level of total gene expression. This value was normalized to the level of *GAPDH *transcripts and expressed relative to that obtained for the heart, which showed a low level of *SPAM1 *expression (Figure 4). As expected, *SPAM1 *was highly expressed in the testis, although low levels of expression were also detected in the prostate and some other tissues.

On the basis of the results obtained with the ORF-specific primers, we decided to quantify the expression of exon 1A and 1B transcripts only in the testis. To avoid amplification of different-sized products from alternatively-spliced *SPAM1 *transcripts, we designed forward primers that spanned two exons. The forward primer for exon 1A transcripts contained 14 bases that anneal to the 3'-end of truncated exon 1A2, and 5 bases that anneal to the 5'-end of exon 3. Similarly, the forward primer for exon 1B transcripts contained 18 bases that anneal to the 3'-end of exon 1B, and 5 bases that anneal to the 5'-end of exon 3. Each forward primer was used with a reverse primer specific to the 3'-end of exon 3. In this way, only the smaller of the two splicing variants originating in each first exon was amplified (see Figure 1A and Table 1). The levels of transcripts detected with these primer pairs were used to calculate the contribution of each ERV-derived promoter to total gene expression. As shown in Figure 4, exon 1A transcripts were approximately 10-fold more abundant than those initiating in exon 1B, accounting for 78% of total *SPAM1 *expression in the testis compared to 7.6% for exon 1B transcripts. The 14% of *SPAM1 *ORF transcripts not accounted for by these primer pairs most likely correspond to splicing variants 1A1 and 1B1, which were excluded from this analysis.

**Table 1:** Primer positions and sequences

Primer name^a^	Exon	Sequence (5'-3')
HGF1	6	CATGAGAAGTATGACAACAGCCTC
HGF2	8	TGGTCTCCTCTGACTTCAAC
HGR1	9	GTTGCTGTAGCCAAATTCGTTGTC
HGR2	9	CTGTAGCCAAATTCGTTGTC
HSF1	4	CTACACTCTATGTGCGCAATCG
HSF2	1A	TAGCAGTGTAGGTGGTTAGCAG
HSF3	1B	GGGTCTAATTGACTTGAGAATGTG
HSF4	4	TTTTTGCATATACCCGCATAG
HSF5	1A-3	TCCTTCCTAGCAAGGGATG
HSF6	1B-3	AAAAAATTAAGCTGAAATGGATG
HSR1	6	TTTGGCTGCTAGTGTGACGTTG
HSR2	4	CTGATGCAAAGTATGAGCACAG
HSR3	4	CATTCCAGGCCCAGAGGAAAG
HSR4	5	CCCATATTACAATTCCAGAAG
HSR5	3	AAGTCTGCTTTCAAAATCCAG
MGF	3	GTGGAGTCTACTGGTGTCTTC
MGR	5	GTGGCAGTGATGGCATGGAC
MSF1	4	GATGCTATGAGTTTAGCACAACG
MSF2	1	ATGATGGAGATGCGAGTGGTAG
MSR1	5	CATCAGATGTCTCCTTACATGTC
MSR2	3	TGTGGTCTGTTTAGTATTAGATGC
MSR3	3	TTCCTTCTTACACACTGTGGTC

a) H, human; M, mouse; G, GAPDH; S, SPAM1; F, forward; R, reverse.

The results obtained by non-quantitative RT-PCR (Figure 3) and quantitative, real-time RT-PCR (Figure 4) were generally similar. However, transcripts containing *SPAM1 *ORF sequence were detected in the small intestine and muscle by the former method, but not the latter. The bands amplified from these tissues by non-quantitative RT-PCR were sequenced and were confirmed to correspond to the predicted *SPAM1 *ORF transcript. 5'-RACE analysis performed on human muscle total RNA identified a low level of transcripts initiating within promoter 1B, but no other *SPAM1*-specific transcripts (data not shown). These results suggest that the 35 cycles used for non-quantitative RT-PCR analysis amplified transcripts present at levels too low to be detected by real-time RT-PCR.

### ERV1-derived promoter 1A is conserved in the mouse genome

**Figure 5:**
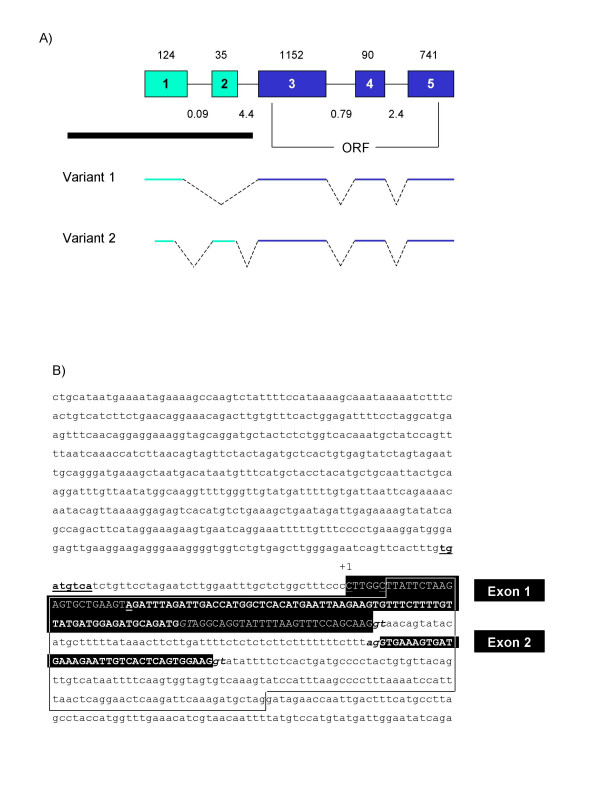
**Genomic structure of murine *Spam1*. **(A) Overview of the *Spam1 *locus. Exons are boxed and numbered, with the size in bp shown above. Intron sizes in kb are given below. The approximate positions of the *Spam1 *ORF start and stop codons are indicated. The two *Spam1 *splicing variants are shown schematically below. ERV-derived sequences are shown in green, with other sequences in blue. The diagram is not to scale. A black bar indicates the region shown in more detail in Figure 5B. (B) Sequence and position of *Spam1 *exons 1 and 2 with respect to TE sequence. The sequence shown corresponds to mouse chromosome 6 co-ordinates 24623802 to 24624821 in the UCSC genome browser (May 2004 release). The solid line frames the sequence annotated as ERV1 MER34 *pol *sequence in the RepeatMasker database. *Spam1 *exon sequences are shown in upper case reverse type. Splicing variant 2 sequences are shown in bold type. The transcription start sites identified by 5'-RACE are underlined; splice donor and acceptor sites are italicized. A non-consensus CRE is shown in bold underlined type.

Initial analysis of *SPAM1 *revealed that transcripts of the mouse orthologue, *Spam1*, also initiate within an ERV [23]. Examination of the mouse genomic sequence revealed that, as in human, the published 5'-ends of the *Spam1 *first exon (nucleotides +12, +21, +48 and +68 in Figure 5B[6,19]) map within an antisense ERV1 MER34 *pol *region (Figure 5B). A similar overlap between *Spam1 *transcripts and an antisense ERV1 *pol *region was observed in the rat genome (data not shown). This ERV1 element therefore inserted into the ancestral genome before the divergence of humans and rodents, approximately 80 million years ago.

**Figure 6:**
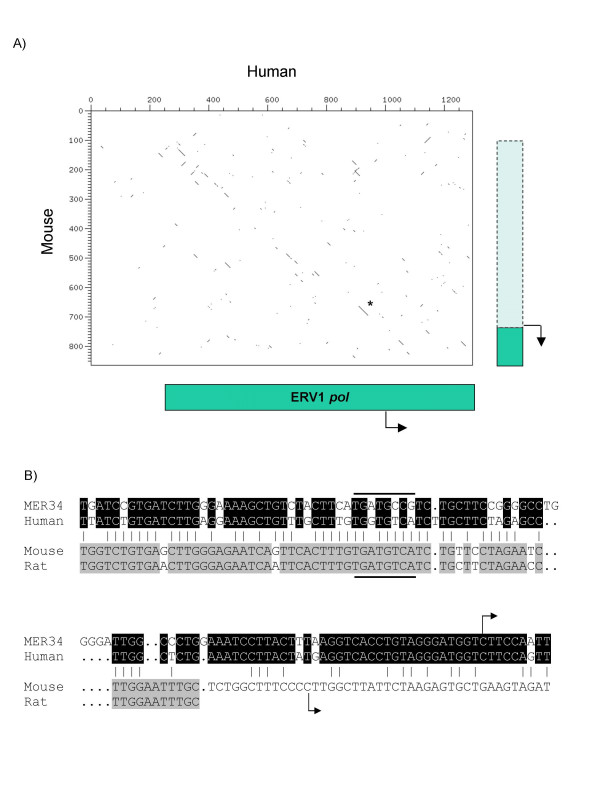
**Comparison of the genomic sequence upstream of the human and mouse *SPAM1 *genes. **(A) DOTTER comparison of the human (horizontal axis) and mouse (vertical axis) genomic DNA sequences upstream of the *SPAM1 / Spam1 *locus. Nucleotide positions in bp are given on the upper horizontal and left vertical axes. The approximate position of the human ERV1 MER34 *pol *region is shown below the lower horizontal axis. The approximate positions of the annotated mouse ERV1 MER34 *pol *region (solid box) and of the extended *pol *sequence (dashed box) are shown on the right hand side. The 5'-most transcriptional start site of each gene is represented by a bent arrow. An asterisk marks the approximate position of a conserved CRE in the proximal human and mouse promoters. (B) Multi-species alignment of the well-conserved sequence marked with an asterisk in Figure 6A. Nucleotides identical between the human and mouse sequence are joined by vertical lines. Nucleotides identical between the mouse and rat sequence are highlighted in gray. The mouse-rat alignment is incomplete in this region due to a small (56 bp) insertion into the rat sequence. The MER34 ERV1 consensus sequence is shown above the human *SPAM1 *promoter sequence; nucleotides identical between the two sequences are shown in reverse type. Solid lines above and below the sequence indicate the position of the conserved CRE. The 5'-most human and mouse transcriptional start sites are marked with bent arrows.

The RepeatMasker track of the UCSC genome browser annotated only a 342 bp region of the murine *Spam1 *locus as ERV1 *pol *sequence; the analogous *pol *fragment containing human *SPAM1 *exon 1A is considerably larger (compare Figure 2 and 5B). Due to the higher neutral mutation rate in mouse [24], murine TEs that inserted prior to the primate-rodent split are roughly twice as diverged as the orthologous human element, making detection by repeat-finding programs difficult. Older elements in rodents are therefore often excluded from annotation as TEs in the RepeatMasker database [24,31]. We used the DOTTER sequence comparison program to determine whether this was the case for the ERV1 sequence associated with *Spam1*. We extracted the human genomic DNA sequence containing the full-length exon 1A1 and 1000 bp of upstream sequence (1296 bp in total) from the UCSC genome browser. We also extracted the mouse genomic sequence containing exon 1 and 734 bp of upstream sequence (a total of 858 bp). A Dot Plot showing a comparison of these two sequences is shown in Figure 6A.

The sequence annotated as the ERV1 *pol *region in the human genome corresponds to nucleotides 246 – 1296 in Figure 6A (nucleotides -754 to +296 in Figure 2). The positions of the ERV1 *pol *region and the exon 1A transcriptional start site are shown below the lower horizontal axis. The mouse genomic sequence from approximately nucleotide 100 – 800 in Figure 6A shows some sequence similarity to nucleotides 300 -1050 of the human sequence. Therefore the region of the mouse *Spam1 *locus derived from the ERV1 *pol *region is considerably larger than that annotated by RepeatMasker, extending approximately 700 bp upstream of the transcriptional start site. The positions of the annotated and extended ERV1 *pol *regions are represented by solid and dashed boxes, respectively, on the right hand side of Figure 6A. A similar DOTTER result was observed upon comparison of the corresponding rat and human genomic sequences (data not shown).

The level of sequence similarity between the human and mouse *SPAM1 *promoter regions is highest at position 900 – 950 in the human sequence and 650 – 700 in the mouse (Figure 6A, region marked with asterisk). A sequence comparison revealed that this conserved region contains the functional CRE identified in the murine *Spam1 *promoter (Figure 6B, reference [19]). The relatively high level of primate – rodent conservation of this element and the surrounding sequence indicates that this region may be functionally important.

We performed 5'-RACE on mouse testis RNA to identify the transcriptional start site(s) and to search for alternative promoters of *Spam1*. As shown in Figure 5, a single first exon with multiple transcriptional start sites was identified for *Spam1*. This exon is orthologous to exon 1A of the human gene (Figure 6A). No sequence equivalent to human exon 1B was detected in mouse *Spam1 *transcripts. Two splicing variants were identified for the mouse *Spam1 *gene. Variant 2 utilized an alternative transcription start site and splice donor site within exon 1 to generate a truncated first exon, and spliced into a short (35 bp) novel downstream exon (Figure 5). As with human *SPAM1*, the murine splicing variants affect only the 5'-UTR, leaving the downstream ORF intact (Figure 5A).

### Expression of the mouse *Spam1 *gene is largely testis-specific

**Figure 7:**
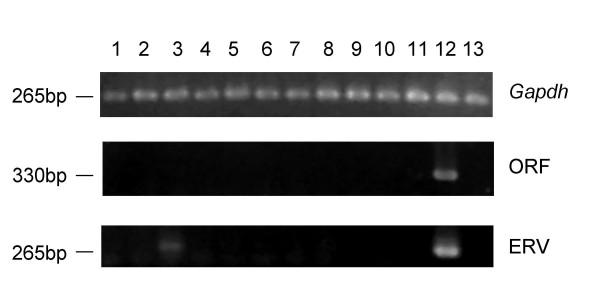
**Detection of *Spam1 *transcripts by RT-PCR. **Primer pairs specific for *Gapdh*, the *Spam1 *ORF, and *Spam1 *transcripts initiating within the ERV sequence were used in RT-PCR assays. The assays were carried out on cDNAs derived from a range of normal C57/BL6 mouse tissues: 1, brain; 2, heart; 3, kidney; 4, liver; 5, lung; 6, colon; 7, small intestine; 8, spleen; 9, stomach; 10, thymus; 11, muscle; 12, testis; 13, placenta. Approximate molecular weights are indicated on the left.

We performed non-quantitative RT-PCR on a panel of normal C57BL/6 mouse tissues to determine the expression pattern of *Spam1*. As shown in Figure 7, primers specific to the *Spam1 *ORF detected transcripts only in the testis. Transcripts initiating within the ERV1 *pol *region were detected primarily in the testis, and to a lesser degree in the kidney. As with the human gene, the ERV1 *pol*-derived promoter of murine *Spam1 *is therefore largely testis-specific.

## Discussion

In this study we have experimentally confirmed a previous *in silico *observation [23] that transcription of the human and murine *SPAM1 *genes initiates within an antisense ERV common to both species. *SPAM1 *is the only hyaluronidase gene to initiate within an ERV (data not shown); this TE insertion therefore took place after the small segmental duplication of three ancestral hyaluronidase genes, but before the divergence of primates and rodents. Interestingly, human *HYAL4*, but not its mouse orthologue, appears to initiate within an antisense LINE1 element ([23] and our unpublished observations). This element therefore inserted after the primate-rodent split, indicating an ongoing contribution by TEs to human hyaluronidase transcriptional regulation.

A previous study by our group determined that TE insertions were more common in transcripts with a high K_a_/K_s _value [23]. The K_a_/K_s _ratio for the human-Old World monkey SPAM1 orthologous pair is high at 0.57 [32]. This is in line with our hypothesis that TE insertions are more likely to be tolerated by rapidly-evolving genes [23]. High K_a_/K_s _ratios are a common characteristic of primate genes that are involved in male reproduction. This may be due to positive selection, driven by competition between the sperm of individual males of the more promiscuous primate species [32]. In the case of SPAM1, the requirement for species-specific sperm-zona pellucida recognition may also have contributed to the high inter-species divergence of the protein sequence.

We have identified two closely-spaced ERV-derived promoters for human *SPAM1*. Both were active primarily in the testis, albeit with an approximately 10-fold difference in promoter activity. This close physical proximity and similar tissue specificity suggests that the two promoters may be regulated by a shared testis-specific enhancer element, rather than by individual tissue-specific proximal promoter regions. We have also identified alternative splicing variants for the human and mouse genes. Alternatively-spliced transcripts of *HYAL1 *and *HYAL3 *have been described that cause an in-frame deletion of the putative catalytic site and abolish hyaluronidase function [33]. Evidence from the NCBI database suggests that an alternative splicing event in *SPAM1 *exon 6 generates an extended 3'-transcript, which encodes a C-terminally truncated SPAM1 isoform. However, the presence of this splicing variant has yet to be confirmed. In contrast, the alternatively-spliced transcripts of *SPAM1 *and *Spam1 *described in this study differ only in the sequence and length of the 5'-UTR, and are not predicted to affect enzyme function. Changes in the 5'-UTR sequence may however alter the stability and / or translation efficiency of the transcripts (reviewed in [34]), and hence impact indirectly on SPAM1 expression.

We have shown that all *SPAM1 / Spam1 *alternative promoter and splicing variants are expressed primarily in the testis. Lower levels of expression were also observed in the human prostate and murine kidney. This contradicts previous reports that human *SPAM1 *is expressed in the placenta [2] and that murine *Spam1 *is expressed in tissues of the female reproductive tract [7]. Expression of SPAM1 is confined to a subset of specialized cells in some tissues [5,7], which may explain these contradictory results.

In contrast to all known examples of host gene transcriptional regulation by ERVs, *SPAM1 *and *Spam1 *initiate not within an LTR, but rather within a fragment of the *pol *coding region. While the *SPAM1 / Spam1 *promoters have not yet been fully analyzed, a non-consensus CRE at position -39 has been shown to be important for activity of the murine *Spam1 *promoter in an *in vitro *testis system [19]. This site, and a similar sequence in the human promoter, are clearly derived from the ERV1 *pol *region and are well conserved between the two species (Figures 2, 5B, and 6). Various lines of evidence suggest that SPAM1 expression is regulated by sex hormones: the expression of SPAM1 in the male and female reproductive organs; the increased expression of *Spam1 *in male kidney compared to female [8]; the seasonal variation in *SPAM1 *expression in red fox testis [35]; and the variations in murine female SPAM1 expression at different stages of estrus [7]. Indeed, various groups have identified putative androgen response elements (AREs) in the *SPAM1 *and *Spam1 *promoters [5,6,19], and estrogen response elements (EREs) in the *Spam1 *promoter [7]. Many of these predicted sites also map within the ERV *pol *region. However, none of these sequences represents a consensus binding site, and none has yet been shown to bind its cognate transcription factor or to be required for SPAM1 expression.

Alternatively, hormonal regulation may be mediated through the CRE. Androgen treatment of Sertoli cells was recently shown to rapidly induce phosphorylation of a CRE binding protein and activate transcription of target genes via the MAPK pathway [36]. This mechanism was postulated to represent a common mechanism for activation of testis-specific promoters that do not contain a consensus ARE. Much work remains to be done to elucidate the mechanisms of transcriptional regulation of *SPAM1 *and *Spam1*. However, it is clear that at least one functional transcription factor binding site is derived from the ERV1 *pol *region.

ERV LTRs contain the regulatory signals necessary for transcription of the retroviral genes. Insertion of an LTR sequence near a host gene could therefore provide a novel, pre-formed regulatory unit and be rapidly adopted by the gene for use as an alternative promoter. It is less clear how a retroviral protein coding region, which has no known function in transcriptional regulation, could be adopted for use as a promoter by a host gene. We suggest the following scenario.

Prior to the primate-rodent divergence, an ERV inserted upstream of the ancestral *SPAM1 *gene, in the antisense orientation. By chance, the antisense *pol *coding region contained sequences that were similar to a CRE, and possibly to other transcription factor binding sites necessary for testis-specific transcription. The region of the human *SPAM1 *promoter that contains the CRE is quite divergent from the MER34 consensus sequence (Figure 6B). It is therefore unlikely that the CRE was functional, and hence preserved by purifying selection, from the time of ERV insertion. The CRE present in the modern *SPAM1 *and *Spam1 *promoters is more likely to have evolved by random nucleotide substitution from a similar sequence in the original antisense *pol *gene. The ~50 bp of genomic sequence that contains the CRE is relatively well conserved between human and rodents (Figure 6), indicating that purifying selection of this sequence probably occurred at some time after the creation of the functional CRE. The evolutionary origins of other functional transcription factor binding sites in the modern *SPAM1 / Spam1 *promoters remain to be determined.

The selective processes driving the evolution of a promoter from a protein coding sequence, and the fate of the original ancestral *SPAM1 *promoter, remain unknown. This gene therefore represents an extremely intriguing example of how the host genome can adopt "parasitic" ERV sequences for its own purposes.

## Conclusion

We have shown that transcription of the human and mouse *SPAM1 *genes initiates within an antisense ERV *pol *gene. The first exons and proximal promoters of both genes are derived from this ancient ERV *pol *sequence. Expression of the human and mouse *SPAM1 *genes is largely testis-specific, and we have provided evidence that testis-specific transcription factor binding sites are derived from conserved ERV sequence in both species. *SPAM1 *can therefore be added to the growing list of mammalian genes that are regulated by TEs. This gene represents the first known example of the evolution of promoter function from an ERV coding sequence, and of gender-specific transcription from an ERV-derived promoter.

## Methods

### Computational methods

The human, mouse and rat *SPAM1 */ *Spam1 *loci were examined using the University of California, Santa Cruz genome browser [37]. Homology searches were performed using the Basic Local Alignment Search Tool (BLAST, [38]). The SPIDEY alignment program [39] was used to compare cDNA and genomic DNA sequences for all splicing variants and for 5'-RACE clones. Human and mouse genomic DNA sequences were compared using the DOTTER program [40].

### Reverse transcription and RT-PCR

C57BL/6 mouse testis total RNA and all human total RNAs were purchased from Clontech. All other mouse RNAs were extracted from C57BL/6 mouse tissues using TRIzol (Invitrogen) according to the manufacturer's instructions. 5 μg of each RNA was treated with DNase I and reverse transcribed as described [41]. 35 cycles of RT-PCR were performed using Taq DNA polymerase with 2 ng/μl of each primer in 4 mM MgCl_2_. Primer pairs were as follows. *GAPDH*, HGF1 & HGR1; *SPAM1 *ORF, HSF1 & HSR1; *SPAM1 *Exon 1A, HSF2 & HSR2; *SPAM1 *Exon 1B, HSF3 & HSR2; *Gapdh*, MGF & MGR; *Spam1 *ORF, MSF1 & MSR1; *Spam1 *ERV, MSF2 & MSR2. All primer positions and sequences are given in Table 1.

### 5'-RACE

5'-RACE analysis of human or mouse testis total RNA was carried out using the FirstChoice RLM-RACE kit (Ambion) as described [42]. HSR3 and HSR2 were used as the outer and inner primers, respectively, for nested RT-PCR amplification of *SPAM1 *5'-RACE products. MSR3 and MSR2 were used as the equivalent mouse primers.

### Real-time RT-PCR

Real-time quantification of transcript levels was carried out as described [42]. Dissociation curves demonstrated that each primer pair amplified a single product. Standard curves were prepared for each primer pair using serial dilutions of human testis cDNA to enable calculation of the relative abundance of each transcript type. The level of *SPAM1 *ORF transcripts for each tissue was normalized to *GAPDH *and expressed relative to the level detected in heart cDNA. The relative amounts of *SPAM1 *ERV1A and ERV1B transcripts were assessed only in testis cDNA. The level of each transcript was divided by the amount of ORF transcript detected in testis. This value was then multiplied by the *GAPDH*- and heart-normalized level of ORF transcripts to determine the contribution of each ERV promoter to total *SPAM1 *expression. Primer pairs were as follows. *GAPDH*, HGF2 & HGR2; Total *SPAM1*, HSF4 & HSR4; *SPAM1 *Exon 1A, HSF5 & HSR5; *SPAM1 *Exon 1B, HSF6 & HSR5.

## List of abbreviations

ARE, androgen response element; CRE, cAMP response element; CREM, CRE modulator; ERE, estrogen response element; ERV, endogenous retrovirus; GAPDH, glyceraldehyde-3-phosphate dehydrogenase; LINE, long interspersed nuclear element; LTR, long terminal repeat; MAPK, mitogen activated protein kinase; ORF, open reading frame; RACE, rapid amplification of cDNA ends; SINE, short interspersed nuclear element; SPAM1, sperm adhesion molecule 1; TE, transposable element; UCSC, University of California at Santa Cruz; UTR, untranslated region.

## Authors' contributions

CAD carried out all experimental work described in the paper, participated in the design of the study, and drafted the manuscript. DLM conceived of the study, participated in its design, and participated in the drafting and critical revision of the manuscript. Both authors read and approved the final manuscript.
